# Automated abnormality classification of chest radiographs using deep convolutional neural networks

**DOI:** 10.1038/s41746-020-0273-z

**Published:** 2020-05-14

**Authors:** Yu-Xing Tang, You-Bao Tang, Yifan Peng, Ke Yan, Mohammadhadi Bagheri, Bernadette A. Redd, Catherine J. Brandon, Zhiyong Lu, Mei Han, Jing Xiao, Ronald M. Summers

**Affiliations:** 1 Imaging Biomarkers and Computer-Aided Diagnosis Laboratory, Radiology and Imaging Sciences, National Institutes of Health Clinical Center, Bethesda, MD 20892 USA; 2 National Center for Biotechnology Information, National Library of Medicine, National Institutes of Health, Bethesda, MD 20894 USA; 3 Clinical Image Processing Service, Radiology and Imaging Sciences, National Institutes of Health Clinical Center, Bethesda, MD 20892 USA; 4 Radiology and Imaging Sciences, National Institutes of Health Clinical Center, Bethesda, MD 20892 USA; 5 Department of Radiology, University of Michigan, Ann Arbor, MI 48109 USA; 6 PAII Inc, Palo Alto, CA 94306 USA; 7 Ping An Technology, Shenzhen, Guangdong 518029 China

**Keywords:** Radiography, Biomedical engineering

## Abstract

As one of the most ubiquitous diagnostic imaging tests in medical practice, chest radiography requires timely reporting of potential findings and diagnosis of diseases in the images. Automated, fast, and reliable detection of diseases based on chest radiography is a critical step in radiology workflow. In this work, we developed and evaluated various deep convolutional neural networks (CNN) for differentiating between normal and abnormal frontal chest radiographs, in order to help alert radiologists and clinicians of potential abnormal findings as a means of work list triaging and reporting prioritization. A CNN-based model achieved an AUC of 0.9824 ± 0.0043 (with an accuracy of 94.64 ± 0.45%, a sensitivity of 96.50 ± 0.36% and a specificity of 92.86 ± 0.48%) for normal versus abnormal chest radiograph classification. The CNN model obtained an AUC of 0.9804 ± 0.0032 (with an accuracy of 94.71 ± 0.32%, a sensitivity of 92.20 ± 0.34% and a specificity of 96.34 ± 0.31%) for normal versus lung opacity classification. Classification performance on the external dataset showed that the CNN model is likely to be highly generalizable, with an AUC of 0.9444 ± 0.0029. The CNN model pre-trained on cohorts of adult patients and fine-tuned on pediatric patients achieved an AUC of 0.9851 ± 0.0046 for normal versus pneumonia classification. Pretraining with natural images demonstrates benefit for a moderate-sized training image set of about 8500 images. The remarkable performance in diagnostic accuracy observed in this study shows that deep CNNs can accurately and effectively differentiate normal and abnormal chest radiographs, thereby providing potential benefits to radiology workflow and patient care.

## Introduction

Cardiothoracic and pulmonary abnormalities are one of the leading causes of morbidity, mortality, and health service use worldwide^1^. According to the American Lung Association, lung cancer is the number one cancer killer of both women and men in the United States, and more than 33 million Americans have a chronic lung disease^2^. The chest radiograph (chest X-ray) is the most commonly requested radiological examination owing to its effectiveness in the characterization and detection of cardiothoracic and pulmonary abnormalities. It is also widely used in lung cancer prevention and screening. Timely radiologist reporting of every image is desired, but not always possible due to heavy workload in many large healthcare centers or the lack of experienced radiologists in less developed areas. Consequently, an automated system of chest X-ray abnormality classification^3,4^ would be advantageous, allowing radiologists to focus more on assessing pathology on abnormal chest X-rays.

Deep learning^5^, a subfield of machine learning, has seen a remarkable success in recent years. It is emerging as the leading machine learning tool in various fields such as computer vision, natural language processing, speech recognition, social media analysis, bioinformatics and medical image analysis^6,7^. In particular, deep convolutional neural networks (CNNs) have proven to be powerful tools for a wide range of computer vision tasks, predominantly driven by the emergence of large-scale labeled datasets and more powerful computational capabilities. CNNs take raw data (e.g., images) as input and perform a series of convolutional and non-linear operations to hierarchically learn rich information about the image, in order to bridge the gap between high-level representation and low-level features. During the training phase, the CNNs adjust their filter values (weights) by optimizing certain loss functions through forward passes and backpropagation procedures, so that the inputs are correctly mapped to the ground-truth labels. Remarkably, CNNs have recently been shown to match or exceed human performance in visual tasks such as natural image classification^8^, skin cancer classification^9^, diabetic retinopathy detection^10^, wrist fracture detection in radiographs^11^, and age-related macular degeneration detection^12^.

Pioneering work in computer-aided diagnosis on chest radiographs mainly focused on a specific disease (e.g., pulmonary tuberculosis classification^13^, lung nodule detection^14^). The recent release of the large-scale datasets, such as “NIH ChestX-ray 14”^15^ (which is an extension of the eight common disease patterns in “NIH ChestX-ray 8”^16^), “CheXpert”^17^ and “MIMIC-CXR”^18^, have enabled many studies using deep learning for automated chest radiograph diagnosis^19,20^. However, the performance of these algorithms is not as good as radiologists for many categories, possibly due to the class-imbalance of the dataset and label noise caused by natural language processing (NLP)^21,22^. Despite all this, a deep convolutional neural network could be trained to identify abnormal chest X-rays with appropriate performance, in order to prioritize studies for rapid review and reporting^4,23^. A recent study^3^ presented a CNN trained and tested on the combination of abnormal radiographs (*n* = 51,760, 97.4%) from the NIH “ChestX-ray 14” database and normal radiographs (*n* = 1389, 2.6%) from the Indiana University hospital network^24^. An area under the receiver operating characteristic curve (AUC) of 0.98 (95% confidence interval (CI): (0.97, 0.99)), a sensitivity of 94.6% and a specificity of 93.4% were reported. However, the normal radiographs were extracted from one hospital while the abnormal ones were from another hospital due to image and label availability, potentially biasing the evaluation (e.g., by classifying based on different qualities or intensities, or even imaging device manufacturers from different hospitals). Moreover, the model was trained and tested on mostly abnormal radiographs which were highly unlikely to represent the real-world prevalence, and was, therefore, unlikely to represent true systematic model inaccuracies. Very recently, Annarumma et al.^23^ used about 0.5 million digital chest radiographs labeled by NLP to train an ensemble of two CNNs to predict the priority level (i.e., critical, urgent, nonurgent, and normal). The sensitivity and specificity of this predictive system for critical abnormalities were 65% and 94%, respectively. Simulations showed abnormal radiographs with critical findings were reviewed sooner by radiologists (2.7 versus 11.2 days on average) with the help of automated priority level prediction compared with actual practice in their institution.

In this paper, we assess the performance of deep CNNs at the task of normal versus abnormal chest X-ray classification. We restrict the comparisons between the algorithms and radiologists to image-based classification. Various deep CNN architectures, e.g., AlexNet^25^, VGG^26^, GoogLeNet^27^, ResNet^28^, and DenseNet^29^ were trained and validated on the training and validation set respectively, and then were evaluated on the test set based on the labels from the attending radiologists and the consensus of three board-certified radiologists, respectively. Receiver operating characteristic curves (ROCs), AUCs, and confusion matrix analysis were used to assess the model performance. Dunnmon et al.^4^ presented a similar system trained and tested on the radiographs from their institution, wherein they achieved an AUC of 0.96 on the normal versus abnormal classification task, and they compared (1) the impact of different CNN architectures for binary classification, (2) the effect of training from scratch and pre-training, (3) the differences between attending radiologist (who read the original scan and composed the text report) and radiologist consensus labels, and evaluated the utility of combining the model prediction with the read of the attending radiologist and performance on different disease sub-types. In the light of ref. ^4^, we additionally evaluate with more CNN architectures, analyze the impact of different image resolutions, and perform external validation to study the generalizability of the model trained from one cohort and applied to another. The results indicate that the deep neural networks achieve accuracy on par with experienced radiologists.

## Results

### Model performance on the NIH “ChestX-ray 14” dataset

**Fig. 1:**
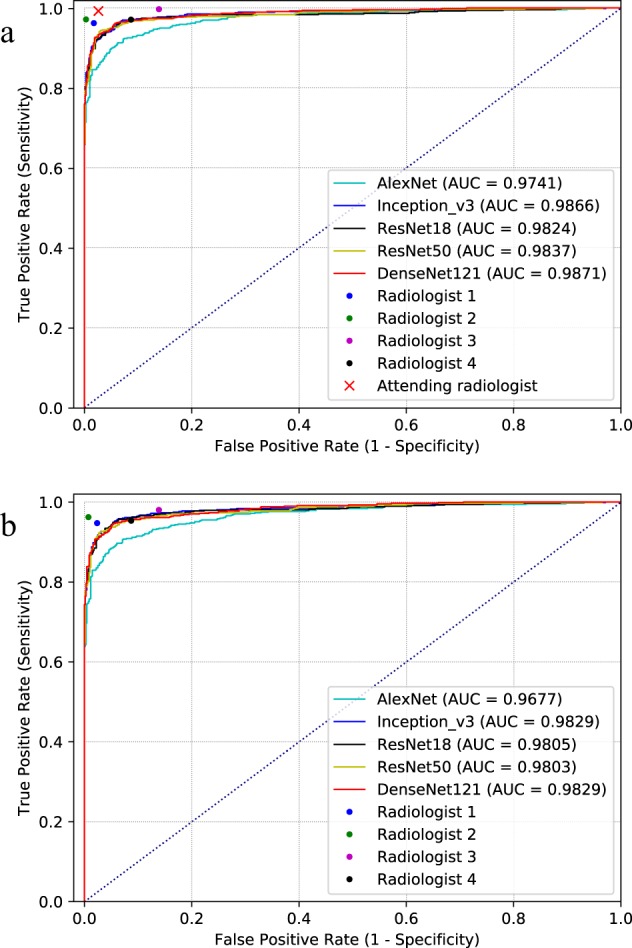
Receiver operating characteristic curves (ROCs) for different ImageNet pre-trained CNN architectures versus radiologists on the NIH “ChestX-ray 14” dataset. **a** Labels voted by the majority of radiologists as the ground-truth reference standard. **b** Labels derived from text reports as the ground-truth reference standard. Radiologists’ performance levels are represented as single points (or a cross for attending radiologist who wrote the radiology report). AUC area under the curve.

**Fig. 2:**
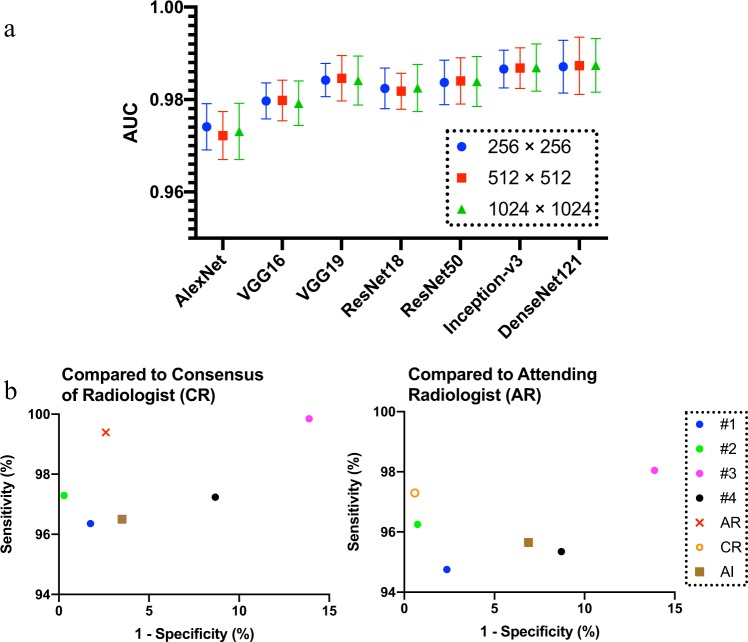
Comparisons of model performance and different radiologists. **a** Performance of different CNN architectures with different input image sizes on the NIH “ChestX-ray 14” dataset. CNN weights were initialized from the ImageNet pre-trained models. Performances are not significantly different among different input image sizes. The error bars represent the standard deviations to the mean values. **b** True positive rate (sensitivity) and false positive rate (1-specificity) of different radiologists (#1, #2, #3, and #4) against different ground-truth labels. Left depicts performance comparisons when setting the consensus of radiologists as ground-truth. Right depicts comparisons when setting labels from attending radiologist as ground-truth. AR attending radiologist, CR consensus of radiologists (vote by the majority of three board-certified radiologists), AI the artificial intelligence model (ResNet18 CNN model shown here).

The consensus labels of three U.S. board-certified radiologists (the majority of votes of Radiologist #1, #2, and #3) were used as the reference standard of “ground truth”. Table 1, Figs. 1a, 2a summarize the performance of different deep convolutional neural networks (such as AlexNet^25^, VGGNet^26^, ResNet^28^, Inception-v3 (GoogLeNet)^27^, and DenseNet^29^), assessed on the test set of the NIH “ChestX-ray 14” dataset (an extension of the “ChestX-ray 8” dataset^16^), using images with a 256 × 256 resolution. All CNNs achieved AUCs higher than 0.96, showing good performance for this binary classification task. The transfer learning method (CNN weights pre-trained on ImageNet^30^) outperformed the models trained from scratch (CNN weights randomly initialized) (*P* < 0.05 (range [0.004, 0.047]) for all the CNN models) with a moderate sized training set of about 8500 images. AlexNet achieved inferior results compared to all other CNN models (*P* < 0.05) and VGG16 achieved inferior results compared to VGG19, Inception-v3, and DenseNet121 (*P* < 0.05). There were no significant differences amongst VGG19, ResNet18, ResNet50, Inception-v3, and DenseNet121 (*P* > 0.05). For instance, ResNet18 (AUC: 0.9824, 95% CI (0.979, 0.986)) achieved a sensitivity/specificity of 96.50/92.86%, an accuracy of 94.64% and an *F*1 score of 0.9463. The positive predictive value (PPV), indicating the probability that the radiograph is abnormal when the prediction is positive, was 92.84%; the negative predictive value (NPV), indicating the probability that the radiograph is normal when the prediction is negative, was 96.52%. As shown in Fig. 2a, AUCs attained with models trained by using input image size 256 × 256, 512 × 512, or 1024 × 1024 pixels were not significantly different. Figure 1 shows the ROC curves of selected CNN models evaluated with reference labels from radiologist consensus (shown in sub figure a) and the attending radiologist (sub figure b).

**Table 1:** Classification performance metrics for different CNN architectures on the NIH “ChestX-ray 14” database.

Models	AUC	Sensitivity (%)	Specificity (%)	PPV (%)	NPV (%)	*F*1 score	Accuracy (%)
AlexNet (P)	0.9741 ± 0.0050	94.18 ± 0.47	87.70 ± 0.56	87.66 ± 0.61	94.10 ± 0.41	0.9091 ± 0.0057	90.85 ± 0.48
AlexNet (S)	0.9684 ± 0.0043	92.65 ± 0.45	87.99 ± 0.41	87.94 ± 0.57	92.68 ± 0.38	0.9023 ± 0.0052	90.25 ± 0.45
VGG16 (P)	0.9797 ± 0.0039	94.03 ± 0.36	90.74 ± 0.41	90.56 ± 0.45	94.14 ± 0.43	0.9226 ± 0.0038	92.34 ± 0.40
VGG16 (S)	0.9742 ± 0.0044	93.42 ± 0.40	91.46 ± 0.46	91.18 ± 0.50	93.63 ± 0.46	0.9228 ± 0.0040	92.41 ± 0.42
VGG19 (P)	0.9842 ± 0.0036	97.09 ± 0.39	87.99 ± 0.35	88.42 ± 0.41	96.97 ± 0.43	0.9255 ± 0.0035	92.41 ± 0.33
VGG19 (S)	0.9757 ± 0.0054	94.49 ± 0.59	88.86 ± 0.49	88.90 ± 0.56	94.46 ± 0.47	0.9161 ± 0.0048	91.59 ± 0.50
ResNet18 (P)	0.9824 ± 0.0043	96.50 ± 0.36	92.86 ± 0.48	92.84 ± 0.55	96.52 ± 0.30	0.9463 ± 0.0041	94.64 ± 0.45
ResNet18 (S)	0.9766 ± 0.0034	96.63 ± 0.41	85.09 ± 0.33	85.97 ± 0.47	96.39 ± 0.36	0.9099 ± 0.0034	90.70 ± 0.38
ResNet50 (P)	0.9837 ± 0.0048	96.94 ± 0.50	88.42 ± 0.61	88.78 ± 0.73	96.83 ± 0.39	0.9268 ± 0.0055	92.56 ± 0.54
ResNet50 (S)	0.9775 ± 0.0057	94.32 ± 0.54	90.59 ± 0.66	90.43 ± 0.75	94.42 ± 0.44	0.9233 ± 0.0059	92.40 ± 0.60
Inception-v3 (P)	0.9866 ± 0.0041	97.38 ± 0.35	87.57 ± 0.48	88.11 ± 0.55	97.26 ± 0.27	0.9250 ± 0.0051	92.33 ± 0.42
Inception-v3 (S)	0.9796 ± 0.0034	95.08 ± 0.32	89.58 ± 0.35	89.58 ± 0.42	95.08 ± 0.23	0.9225 ± 0.0047	92.25 ± 0.37
DenseNet121 (P)	0.9871 ± 0.0057	97.40 ± 0.53	87.55 ± 0.68	88.09 ± 0.74	97.27 ± 0.33	0.9251 ± 0.0056	92.34 ± 0.56
DenseNet121 (S)	0.9801 ± 0.0044	95.10 ± 0.38	90.01 ± 0.49	90.00 ± 0.61	95.11 ± 0.27	0.9248 ± 0.0041	92.49 ± 0.44

CNN model predictions were compared with the consensus labels of three board-certified radiologists.

*AUC* area under the receiver operating characteristic curve, *PPV* positive predictive value (or precision), *NPV* negative predictive value.

P: model weights were initialized from the ImageNet pre-trained model. S: random initialization of model weights, i.e., training from scratch.

### Model performance compared with radiologists on the NIH “ChestX-ray 14” dataset

The average time for the readers to manually label the 1344 radiographs was 2.3 h (time range: 1.5–3.2 h, coarsely accounted according to software use time, long idle time not accounted). The interrater agreement between the consensus of three U.S. board-certified radiologists (the majority of votes of Radiologist #1, #2, and #3) and the attending radiologist (who read the original scan and composed the text report) was 98.36%, with a Cohen *κ* score of 0.9673. This implies a “perfect” agreement between the labels from the attending radiologist (first extracted using NLP and then corrected by manually checking with the report) and the expert consensus. The interrater agreement between the initial automated NLP labels extracted from radiology reports and the expert consensus was 96.95% (*κ* = 0.9390), showing good but inferior results than manual labeling based on the report. The interrater agreement between readers was 94.83 ± 2.27%, with a Cohen *κ* score of 0.8966 ± 0.045. Sensitivity and specificity of different radiologists (#1, #2, #3, and #4) using the consensus of three board-certified radiologists (CR) as ground-truth reference standard are shown in Figs. 1a and 2b-left. The results using the labels from the attending radiologist (AR) as reference are shown in Figs. 1b and 2b-right.

### Model performance on the RSNA pneumonia detection challenge dataset

**Fig. 3:**
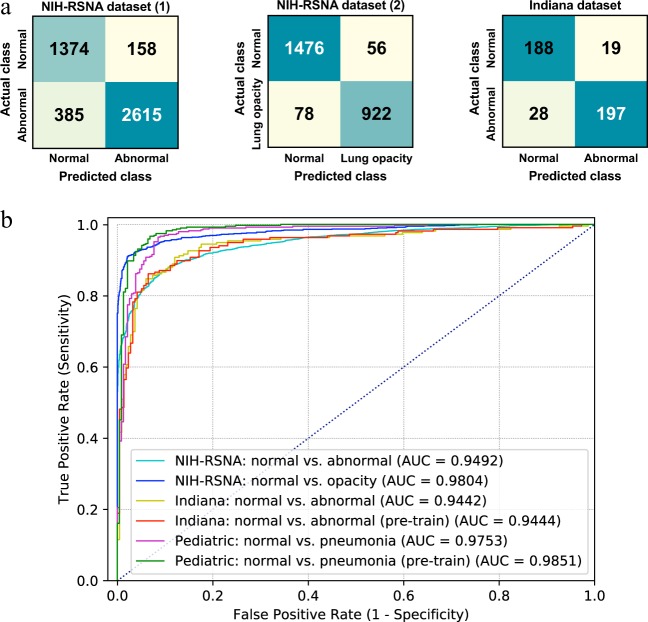
Model performance on different datasets and tasks. **a** Confusion matrices of VGG-19 CNN model performance on different datasets. Left: RSNA challenge dataset for normal versus abnormal classification. Middle: RSNA challenge dataset for normal versus lung opacity classification. Right: Indiana dataset for normal versus abnormal classification. **b** ROCs of CNN models’ performance on different datasets and tasks. Pre-train: CNNs pre-trained on the NIH “ChestX-ray 14” dataset for normal versus abnormal classification as weight initialization.

We first trained a normal versus abnormal (pneumonia-like and other forms of lung opacity) CNN classifier and performed seven-fold cross-validation on 21,152 chest radiographs (normal = 6993, 33.06%; abnormal = 14,159, 66.94%). The CNN model was VGG-19 since we observed that there was no significant difference amongst different models except AlexNet according to our previous experiments. The model was tested on a hold-out test set of 4532 radiographs (normal = 1532, 33.80%; abnormal = 4532, 66.20%). The AUC was 0.9492 (95% CI [0.9441, 0.9550]), sensitivity was 87.17% and specificity was 89.69%. The positive predictive value was 94.30% and the negative predictive value was 78.11%. We then trained a normal versus pneumonia-like lung opacity VGG19 classifier and performed seven-fold cross-validation on 11,652 chest radiographs (a subset of the first experiment on this dataset. Normal = 6993, 60.02%; abnormal with lung opacity = 4659, 39.98%). The test set contains 2532 radiographs (normal = 1532, 60.51%; abnormal with lung opacity = 1000; 39.49%). An AUC of 0.9804 (95% CI [0.9771, 0.9838]) was achieved, sensitivity was 92.20% and specificity was 96.34%. The positive predictive value was 94.27% and negative predictive value as 94.98%. These imply that the automated system is competent to the task of differentiating pneumonia radiographs from normal ones. The confusion matrices and ROCs of the model are shown in Fig. 3.

### Model performance on the Indiana dataset

Firstly, we applied the VGG19 model trained on the NIH “ChestX-ray 14” dataset to predict on 432 chest radiographs of the Indiana test set. An AUC of 0.9442 was obtained, with a sensitivity of 92.59% and specificity of 83.33%. Since the image and patient distribution of the NIH dataset and the Indiana dataset might be different, we then fine-tuned this VGG19 model on 3381 radiographs on the latter and applied the fine-tuned model on the same 432 test images. We obtained an AUC of 0.9444, with a sensitivity of 87.04% and specificity of 91.20%. We did not observe a significant difference by fine-tuning on the target dataset in this task. This suggests that the model trained on the large NIH dataset can generalize well to the Indiana dataset, probably because only limited domain shift exists between these two datasets. The ROCs of both models and the confusion matrix of the fine-tuned model are shown in Fig. 3.

### Model performance on the WCMC pediatric dataset

We used GoogLeNet (Inception-v3) model for this task since it was statistically equivalent to other models but was chosen as an example. The GoogLeNet model trained on the NIH adult chest radiographs for normal versus pneumonia classification (adult model), obtained an AUC of 0.9160 in the test set of pediatric data of WCMC. The same CNN architecture achieved an AUC of 0.9753 when trained using the pediatric radiographs from the training set of WCMC. This indicated a significant domain shift between these two patient cohorts. We observed a performance improvement when the pre-trained adult model was being fine-tuned on the pediatric data. This hybrid model achieved a high AUC of 0.9851 classifying normal and pneumonia pediatric chest radiographs. The ROCs are shown in Fig. 3b.

### Visualization of the deep learning model

**Fig. 4:**
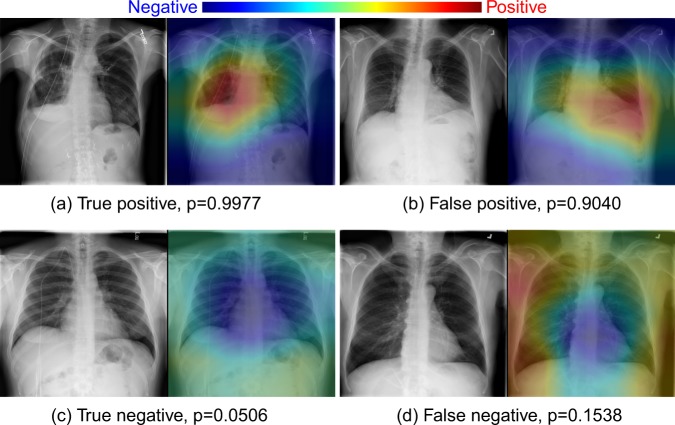
Model visualization. For each group of images, left is the original radiograph, right is the heatmap overlaid on the radiograph. The areas marked with a peak in heatmap indicate the prediction of abnormalities with high probabilities. **a** Findings: right lung pneumothorax. All four radiologists labeled it as abnormal. CNN model predicts it as abnormal with a confidence score of 0.9977. **b** Impression: no evidence of lung infiltrate thoracic infection. Two of four radiologists labeled it as normal, the other two labeled it as abnormal. Model prediction: abnormal, score: 0.9040. **c** Impression: normal chest. All four radiologists labeled it as normal. Model prediction: normal, abnormality score: 0.0506. **d** Findings: minimally increased lung markings are noted in the lung bases and perihilar area consistent with fibrosis unchanged. Two of four radiologists labeled it as abnormal, the other two labeled it as normal. Model prediction: normal, score: 0.1538. Findings and impressions were extracted from the associated text reports.

To aid interpretation of the results toward model transparency, we show some selected examples (true positive, false positive, true negative, and false negative) of model visualization, i.e., the activation of the ResNet18 model in a spatial extent on top of the radiographs using class activation maps^4,16,31^ in Fig. 4. These examples suggest that the CNN model also has the potential to focus on clinically meaningful abnormal regions of the chest radiographs for the classification task that trained only with labels indicating the presence or absence of abnormality.

## Discussion

Here we demonstrate the effectiveness of deep convolutional neural networks in classifying normal and abnormal chest radiographs. A single best convolutional neural network trained on a moderate-sized (approximately 8500 radiographs) dataset with moderate image resolution (256 × 256 pixels), achieves a high AUC of 0.98, with a sensitivity of 96.50% and specificity of 92.86% (i.e., ResNet18). It is able to match the performance of radiologists in this binary classification task, on the testing radiographs sourced from the same institution as the training chest radiographs, with significantly less inference time (50 s for a deep learning network versus 2.3 h for radiologists on average for 1344 chest X-rays). Additionally, in general, the choice of deep CNN architectures did not influence the overall classification performance. Deeper networks tend to work better at classifying more categories^16^, or for more sophisticated tasks such as detection or segmentation. Deeper networks did not show significant improvement when the number of convolutional layers increased for this specific binary classification task. Using a training set of about 8500 images, we found that the ImageNet pre-training outperformed training from scratch. This is consistent with Dunnmon et al.^4^, where ResNet-18 model pretrained from ImageNet outperforms the same model trained from scratch using 18,000 training chest X-ray images and 200 validation images (AUC 0.94 versus 0.90). However, in Dunnmon et al.^4^, training with 180,000 chest X-ray images did not show significant differences between pretraining and training from scratch. Consequently, pretraining could be more beneficial to a moderate sized dataset (e.g., about 8500 images in the NIH dataset or 18,000 images in Dunnmon et al.^4^) than a sufficiently large dataset (e.g., 180,000 images in Dunnmon et al.^4^).

The binary labels of the NIH training set were obtained by natural language processing. Such labels are considered to represent weak (or “noisy”) supervision in the training process. Although deep CNNs were trained with “noisy” labels, the performance in the test stage where ground-truth labels were available shows their robustness in handling label noise. These findings align with a previous study^4^, where the CNNs were trained using a larger number of radiographs. The appearance and statistical properties of chest radiographs are affected by scanner technology, acquisition settings, and post-processing techniques^32^. In this retrospective study, there were various scanner types from different manufacturers and recording settings in the National Institutes of Health Clinical Center, from where the NIH “ChestX-ray 14” dataset was constructed. Therefore, this dataset covers a sufficiently large variability in chest X-ray appearances. Additionally, to further increase variability, training images were randomly gone through pixel-level transformations (including contrast and brightness adjustment) and spatial-level transformations (scale shift and rotation). When deploying the trained model on the NIH dataset to the external dataset (Indiana dataset), it also achieved good classification (AUC = 0.94), demonstrating the generalizability of deep learning models under limited domain shift.

For the RSNA pneumonia detection dataset, labels were purely manual annotated by radiologists based only on the image appearances. While for the NIH “ChestX-ray 14” dataset, labels were extracted from the text reports. However, the results on these two different datasets are not directly comparable because: (1) different numbers of training images existed in these two datasets, (2) different ratios of normal/abnormal images exist both in the training and the testing set. We reported the empirical results on the publicly available RSNA dataset of different labeling manner than NLP.

In Rajpurkar et al.^21^, they trained a DenseNet121 on the NIH “ChestX-ray 14” dataset and evaluated the model for pneumonia recognition on a subset of the NIH “ChestX-ray 14” testing set (420 images). Their results are not directly comparable to ours on the NIH-RSNA dataset since they classified chest X-rays as either with pneumonia or without pneumonia, while we classified X-rays as either normal or pneumonia-related lung opacity. Moreover, they used the same NLP labels as Wang et al.^16^, for training, but they re-labeled the testing set with radiologists. In contrast, we used radiologist labels for both training and testing. Most importantly, the definition of “pneumonia” was essentially different in Wang et al.^16^ and Rajpurkar et al.^21^ than the NIH-RSNA dataset as we discussed in the dataset description.

A common criticism of deep learning models in radiology is that they frequently suffer from generalization issues due to large source and target domain divergence. We observed that the harmful effects of domain divergence can be mitigated when transferring knowledge from a source domain (adult chest radiographs) to a target domain (pediatric chest radiograph) by fine-tuning using a small number of labeled images from the target domain. This transfer learning process learns the common characteristics of both domains leading to a better initialization of the model parameters and faster training.

There are several limitations to this study. First, the experiment was a retrospective one, where the labels of the training images were text mined from the radiological report using NLP. A comparison of manual ground-truth labels versus NLP labels would be interesting but unrealistic due to the unavailability of annotation from experienced radiologists for such a large training set. Second, in the reader study, radiologists were provided only with frontal view 1024 × 1024 images in PNG format through a customized tool for annotation. However, in their routine clinical work, they conduct the reporting using a picture archiving and communication system (PACS) that displays digital imaging and communications in medicine (DICOM) images, often with both frontal and lateral views, comparisons to prior imaging studies (such as chest X-rays, CT scans), and other information (such as patient history, lab results). Hence, the performance of labelers in practice may not be consistent with those attained in a controlled environment^4^. Nevertheless, in this binary labeling task, we did not find a significant discrepancy between the two labels sets (a Cohen *κ* score of 0.9673). Even more, the automated NLP labels extracted from the radiology reports showed good agreement with expert consensus (*κ* = 0.9390) on the testing set of the NIH dataset.

As a proof of concept, we focused our evaluations on normal versus abnormal (or pneumonia-like lung opacity) classification in chest radiographs. This study shows that deep learning models offer the potential for radiologists to use them as a fast binary triage tool thus improving radiology workflow and patient care. In addition, this study may allow for future deep learning studies of other thoracic diseases in which only smaller datasets are currently available. Taken together, we expect this study will contribute to the advancement and understanding of radiology and may ultimately enhance clinical decision-making.

## Methods

Our study was compliant with the Health Insurance Portability and Accountability Act and was conducted with approval from the National Institutes of Health Institutional Review Board (IRB) for the National Institutes of Health (NIH) data (Protocol Number: 03-CC-0128, Clinical Trials Number: NCT00057252), and exemption from IRB review for Indiana and Guangzhou datasets. The requirement for informed consent was waived.

### Datasets

We studied three different databases. 1. National Institutes of Health Database: two subsets were used from this database: (a) NIH “ChestX-ray 14” dataset: A total of 112,120 frontal-view chest radiographs and their corresponding text reports were obtained retrospectively from the clinical PACS database at the NIH Clinical Center. We text-mined the radiological reports using the same Natural Language Processing (NLP) techniques used in the ref. ^16^. The abnormalities of major abnormal cardiac and pulmonary findings in this dataset include cardiomegaly, lung opacity (including pneumonia, consolidation, and infiltrate), mass, nodule, pneumothorax, pulmonary atelectasis, edema, emphysema, fibrosis, hernia, pleural effusion, and thickening. These abnormalities were binned into the “abnormal” category, and negative studies were included in the “normal” category. Note that the patients with medical devices (e.g., chest tubes, central venous catheters, endotracheal tubes, feeding tubes, and pacemakers) or healed rib fractures but without any other chest abnormalities were categorized into the “normal” category. We approximately balanced the “normal” and “abnormal” categories (about 50% for each category) to ease the training and evaluation procedures. After automated NLP mining, a total number of 11,596 radiographs were obtained, among which 10,252 were separated into training and validation sets and 1344 for hold-out testing. The labels for the training and validation sets were obtained using only the automated NLP tool, while two different sets of labels were obtained for the testing set. The first set of labels were obtained by using the same NLP tool as above and then corrected by an expert based on the radiology reports. More specifically, a “human in the loop” manual correction process was applied on the 1344 testing images and reports. This process was adopted to correct some potential wrong labels extracted using NLP, from the text reports composed by the attending radiologists. In this process, a human observer (Y.X.T.) checked the label consistency between the binary NLP label and the impression (conclusion) of the attending radiologist, which indicates if a chest X-ray is normal or abnormal in the text report. If there was a discrepancy, a radiologist (M.B.) read the text report and drew conclusion (normal or abnormal). 33 images were sent to the radiologist and 26 of them were eventually corrected by the radiologist. This indicates that the accuracy of NLP on the binary labeling task is 98.07%. This is the so-called “attending radiologist label set”. The other set of labels was obtained by taking the consensus of three US board-certified radiologists. This is denoted as “consensus of radiologists label set”. 677 images were labeled as normal and 667 images were labeled as abnormal by the attending radiologist, while 691 images were labeled as normal and 653 were labeled as abnormal by the consensus of three radiologists. We perform seven-fold cross-validation (about 8500 images for training and the rest for validation) and report the mean and standard deviation results in this experiment. (b) RSNA pneumonia detection challenge dataset: a total of 25,684 chest radiographs from the NIH database were re-labeled by six board-certified radiologists from the Radiological Society of North America (RSNA) and two radiologists of the Society of Thoracic Radiology (STR) into three categories: normal (*n* = 8525, 33.2%), abnormal with lung opacity (*n* = 5659, 22.0%) and abnormal without lung opacity (*n* = 11,500, 44.8%). The definition of “pneumonia-like lung opacity” includes findings like pneumonia, infiltration, consolidation, and other lung opacities that radiologists considered as pneumonia-related. The details of the dataset and annotation process can be found in the ref. ^33^. 2. Indiana University Hospital network database: we used the chest radiographs from the Indiana University hospital network publicly available at the Open-i service of the National Library of Medicine. This dataset contains chest radiographs obtained in both the frontal and lateral projections. We trained an automated tool (available at https://github.com/rsummers11/CADLab) to classify the two views and filtered 3813 de-identified frontal chest radiographs, among which 432 (50% normal, 50% abnormal) were used for testing. The remaining radiographs were used to fine-tune the model trained on the NIH “ChestX-ray 14” dataset. 3. Guangzhou Women and Children’s Medical Center Pediatric Database: a database from Guangzhou Women and Children’s Medical Center (WCMC) in China containing 5856 pediatric chest radiographs were made publicly available by Kermany et al. ^34^. Chest radiographs in this database were either labeled as normal or pneumonia (caused by virus or bacteria). We used the same data split as in the ref. ^34^, where 5232 (1349 normal, 3883 pneumonia) images were used for training and validation, and the remaining 624 (234 normal, 390 pneumonia) radiographs were used for testing.

### Deep convolutional neural network structure and development

We trained various well-known deep CNN architectures such as AlexNet^25^, VGGNet^26^, Inception-v3 (GoogLeNet)^27^, ResNet^28^, and DenseNet^29^. The weights (or parameters) of these models were either pre-trained on about 1.3 million natural images of 1000 object classes from the ImageNet Large Scale Visual Recognition Challenge database^30^ (the so-called “transfer learning” strategy) or randomly initialized (the so-called “training from scratch” strategy). We replaced the final classification layer (1000-way softmax) of each pre-trained CNN with a single neuron with sigmoid operation that outputs the approximate probability that an input image is abnormal. We resized each input chest radiograph to 256 × 256, cropped 224 × 224 center pixels (for Inception-v3, we resized the image to 342 × 342 and cropped 299 × 299 center pixels in order to make it compatible with its original dimensions), and fed them to each individual CNN model. We also evaluate with different input radiograph sizes such as 512 × 512 (448 × 448 crop) and 1024 × 1024 (896 × 896 crop) pixels. CNN models were trained using backpropagation on an NVIDIA TITAN X Pascal graphics processing unit (GPU) with 12 GB memory for 256 × 256 images and on an NVIDIA TITAN V-100 GPU with 32 GB memory for 512 × 512 and 1024 × 1024 images. The loss function was binary cross-entropy loss. We used a grid search to find optimal hyperparameters (learning rate, batch size, etc.). All the layers of the ImageNet pre-trained CNN models were fine-tuned using an initial learning rate [0.0005, 0.001, 0.05, and 0.1] ([0.005, 0.01, 0.05, and 0.1] for models with random initialization) with a weight decay rate of 0.0001, using the stochastic gradient descent (SGD) optimizer with the momentum of 0.9. The learning rate was reduced by a factor of 0.1 after the loss plateaued for five epochs. Early stopping was used to avoid overfitting on the training set with a maximum running of 50 epochs. The batch size was [64, 128] for an image size of 256 × 256, [16, 32] for 512 × 512 and [4, 8] for 1024 × 1024. We empirically found for 256 × 256 input images and a batch size of 64, the optimal learning rate was 0.001 for ImageNet pre-trained models and 0.01 for models with random initialization. We augmented the dataset in the training stage by horizontally flipping the chest radiographs. We implemented the networks using the open-source PyTorch (https://pytorch.org/) deep learning framework.

### Reader study

Four radiologists (Radiologist #1, #2, and #3 are US board-certified, Radiologist #4 is a foreign-trained radiologist) served as human readers to label the same NIH “ChestX-ray 14” test set above. They had a mean of 29.75 years of experience (range 29–31 years). Annotation was performed by using a customized graphical user interface (GUI)-based annotation software installed on readers’ personal computers. The readers were shown chest X-rays in Portable Network Graphics (PNG) format with an image size of 1024 × 1024 pixels; they were able to zoom in and out using the software. The readers were provided with the same guidelines to the annotation software and rules. They were to make binary decisions on the 1344 chest radiographs and were blinded to the text report composed by the attending radiologist who read the original scan and other readers’ annotations. The ratio of normal to abnormal radiographs was not revealed to the readers.

### Quantification and statistical analysis

The predictive performance of the deep CNN models was compared with that of practicing radiologists. We performed seven-fold cross-validation on the training and validation subsets and averaged outputs (scores) of seven models on the test set. The performance metrics were the AUC, sensitivity, specificity, positive predictive value (PPV), negative predictive value (NPV), *F*1 score, accuracy, and confusion matrix. The 95% confidence intervals (CI) were obtained using seven-fold cross-validation. Cohen’s kappa coefficient^35^ was used to assess the inter-rater agreement. These measurements were computed using scikit-learn (https://scikit-learn.org), a free software machine learning library for the Python programing language (https://www.python.org/). The ROC curves were plotted using matplotlib (https://matplotlib.org/), a plotting library for Python. Note that the computer program gave an approximate probability that a chest radiograph was abnormal, while the radiologist only provided a binary (normal or abnormal) decision on a chest radiograph. We set a hard threshold to 0.5 to determine the binary decision of the computer program when required in computing the metrics. Comparisons between AUCs were obtained by using a nonparametric approach^36^, where multiple replicates of each model were trained and tested. We used a *t*-test, provided by the *ttest_ind* function in SciPy (https://www.scipy.org/), an open-source Python library for scientific computing and technical computing, for the statistical test, with a *P*-value less than 0.05 indicating statistical significance. Qualitative results were visualized by highlighting the image regions that were most responsible for the deep CNN classification model using class activation maps^4,16,31^.

## Supplementary information

Supplementary Information

## Data Availability

The NIH chest radiographs that support the findings of this study are publicly available at https://nihcc.app.box.com/v/ChestXray-NIHCC and https://www.kaggle.com/c/rsna-pneumonia-detection-challenge. The Indiana University Hospital Network database is available at https://openi.nlm.nih.gov/. The WCMC pediatric data that support the findings of this study are available in the identifier 10.17632/rscbjbr9sj.3.

## Code availability

Codes are available at https://github.com/rsummers11/CADLab/tree/master/CXR-Binary-Classifier.
